# Mortality and Factors Associated with Death in Children with Congenital Gastrointestinal Malformations—A Five-Year Retrospective Study from Romania (2020–2024)

**DOI:** 10.3390/children13080976

**Published:** 2026-07-23

**Authors:** Iulia Stratulat-Chiriac, Raluca Ozana Chistol, Elena Țarcă, Lăcrămioara Perianu, Viorel Țarcă, Alina Mariela Murgu, Solange Tamara Roșu, Ioana-Alina Halip, Valeriu Chisălău, Cristina Furnică

**Affiliations:** 1 Grigore T. Popa University of Medicine and Pharmacy, 700115 Iasi, Romania; chiriac.iulia@d.umfiasi.ro (I.S.-C.); raluca-ozana.chistol@umfiasi.ro (R.O.C.); tarca.elena@umfiasi.ro (E.Ț.); lacramioara.perianu@umfiasi.ro (L.P.); alina.murgu@umfiasi.ro (A.M.M.); alina-ioana.grajdeanu@umfiasi.ro (I.-A.H.); cristina.furnica@umfiasi.ro (C.F.); 2 Faculty of Medicine, Apollonia University, Strada Pacurari nr. 11, 700511 Iasi, Romania; viorel.tarca@univapollonia.ro; 3 “Piatra Neamt” County Emergency Hospital, 610136 Piatra Neamt, Romania; valeriuchisalau@gmail.com

**Keywords:** congenital gastrointestinal malformations, neonatal surgery, mortality, risk factors, prematurity, sepsis

## Abstract

**Background/Objectives**: Congenital gastrointestinal malformations (CGIMs) remain a significant cause of pediatric mortality, particularly during the neonatal period in resource-limited settings. Mortality varies widely, across conditions and healthcare systems, yet data from Eastern European centers remain limited. We aimed to determine mortality during index admission and identify baseline factors associated with death among children with CGIMs treated at our tertiary surgical center, including the COVID-19 pandemic years. **Methods**: We conducted a single-center retrospective study (January 2020–December 2024) of all consecutive children admitted with congenital gastrointestinal malformations. The primary outcome was death during index admission. Secondary mortality outcomes included death within 30 days of admission and 30-day postoperative mortality. Comparisons were performed between survivors and non-survivors. Data were extracted from medical records and potential risk factors of mortality were evaluated using univariable analyses and multivariable logistic regression. Statistical analysis was performed using SPSS version 31 (IBM) and a *p*-value of <0.05 was considered statistically significant. **Results**: Overall, index-admission mortality was 11.7% (27/231), with 55.6% of deaths occurring within 30 days from admission and 85.2% occurring postoperatively. Mortality was highest among patients with cloacal malformations, duodenal atresia/stenosis, and esophageal atresia ± tracheoesophageal fistula. Deaths occurring during the neonatal period accounted for 85% of all deaths. Compared with survivors, non-survivors had significantly lower birth weight, gestational age, and Apgar scores, increased ASA score, and higher rates of prematurity, syndromic conditions, associated anomalies, and sepsis. In multivariable analysis, prematurity remained independently associated with index-admission mortality (adjusted OR 3.20, 95% CI 1.23–8.31; *p* = 0.017). In an exploratory multivariable analysis including in-hospital complications, sepsis was strongly associated with mortality (adjusted OR 8.63, 95% CI 3.16–23.55, *p* < 0.001). **Conclusions**: Mortality during index admission remained substantial among children with congenital gastrointestinal malformations. Prematurity was the only baseline characteristic independently associated with mortality. In an exploratory analysis, sepsis identified from retrospective clinical documentation was associated with mortality. Improved care for preterm infants and prevention of severe infection may improve outcomes. Larger multicenter studies are needed to validate these findings and refine risk stratification strategies for high-risk patients.

## 1. Introduction

Congenital gastrointestinal malformations (CGIMs) are typically diagnosed in the neonatal period and often require prompt surgical treatment to prevent life-threatening complications [1,2]. This group includes a broad spectrum of conditions such as esophageal atresia, intestinal atresias, anorectal malformations, Hirschsprung disease, and omphalomesenteric duct remnants, which vary in severity and clinical course. Although prenatal detection of congenital gastrointestinal anomalies has improved over time, accurate prediction of postnatal disease severity remains challenging [3]. Timely surgical intervention is often definitive and may reduce short- and long-term morbidity and mortality [4].

Despite substantial advances in neonatal and surgical care over recent decades, CGIMs remain an important cause of pediatric mortality, accounting for an estimated 47,000 deaths globally each year [2,5,6]. Mortality is approximately two- to three-fold higher in low- and middle-income countries than in high-income settings, reflecting persistent disparities in access to timely diagnosis, specialized surgical care, and postoperative support [7,8]. The highest mortality rates are reported in jejuno-ileal atresia, ranging from 5 to 15% in high-income countries to 20–50% in low-income settings [9,10].

In addition, morbidity and mortality are influenced by patient-related factors, associated comorbidities, particularly prematurity, low birth weight, and major congenital cardiovascular anomalies, and postoperative complications [2,11,12]. The risk of death increases dramatically with the number and severity of complications, particularly when multiple organ systems are involved [8].

Evidence from Eastern Europe remains limited, with most comparative data reported at the broader European or income-group level [2,13,14]. This gap highlights the need for evidence from tertiary referral centers to better characterize local mortality patterns and associated risk factors. Therefore, this study aimed to evaluate in-hospital mortality among children with CGIMs treated at a tertiary referral center in Romania between 2020 and 2024. The primary objective was to estimate mortality during index admission, while the secondary exploratory objectives were to identify risk factors associated with death. Index-admission mortality reflects disease severity at presentation, perioperative stabilization, and surgical care, whereas deaths occurring during subsequent admissions may represent fundamentally different clinical processes related to chronic morbidity, intestinal failure, staged surgical management, or long-term complications. Based on previous evidence, we expect mortality to be associated with prematurity, low birth weight, and associated anomalies, while exploratory analyses also examined the contribution of complications during admission [15,16,17].

By providing contemporary regional data, this study may contribute to evidence-informed clinical practice, healthcare planning, and efforts to improve outcomes for children with congenital gastrointestinal malformations in Romania.

## 2. Materials and Methods

### 2.1. Study Design and Setting

This study was a retrospective, observational cohort involving all consecutive patients younger than 18 years diagnosed with at least one congenital gastrointestinal malformation and admitted to the Emergency Hospital for Children “Saint Mary”, in Iasi, Romania, between 1 January 2020 and 31 December 2024.

The hospital serves as the primary surgical tertiary pediatric referral center serving the northeast region of the country and has no inborn obstetric service. Consequently, all neonates admitted were transferred from external maternity services. The cohort partially overlaps with a previously published descriptive study evaluating diagnostic spectrum, prenatal suspicion, and early outcomes of congenital gastrointestinal malformations; however, the present analysis specifically focused on mortality during index admission and risk factors associated with mortality [18]. Core methods for case identification and cohort assembly followed the methodology described in the previous report. The study was conducted and reported in accordance with the Strengthening the Reporting of Observational Studies Epidemiology (STROBE) guidelines [19].

### 2.2. Eligibility Criteria

All children younger than 18 years admitted between January 2020 and December 2024 with a confirmed congenital gastrointestinal malformation were eligible for inclusion.

We excluded isolated abdominal wall defects, congenital diaphragmatic hernia with secondary malrotation, congenital anomalies of the liver, biliary tree and pancreas, acquired gastrointestinal conditions, incomplete surgical data, or cases treated surgically in another institution (except diagnostic biopsy for Hirschsprung disease).

The congenital gastrointestinal malformations in the final analysis were: esophageal atresia ± tracheoesophageal fistula (Q39.0–Q39.2), congenital hypertrophic pyloric stenosis (Q40.0), duodenal atresia/stenosis (Q41.0), malrotation with/without volvulus (Q43.3), intestinal atresia/stenosis (Q41.1—jejunal, Q41.2—ileal, Q41.8—ileo-jejunal), omphalomesenteric duct remnants (Q43.0, most commonly Meckel’s diverticulum), Hirschsprung disease (Q43.1), anorectal malformations (Q42.0–Q42.3), cloacal malformations (Q42.7), duplication cyst (Q43.4), and congenital gastric volvulus (Q40.2). Primary gastric volvulus (Q40.2) was included only when intraoperative findings confirmed congenital hyperlaxity or absence of gastric ligaments, in the absence of acquired causes like prior surgery, adhesions or trauma.

### 2.3. Data Collection

**Figure 1:**
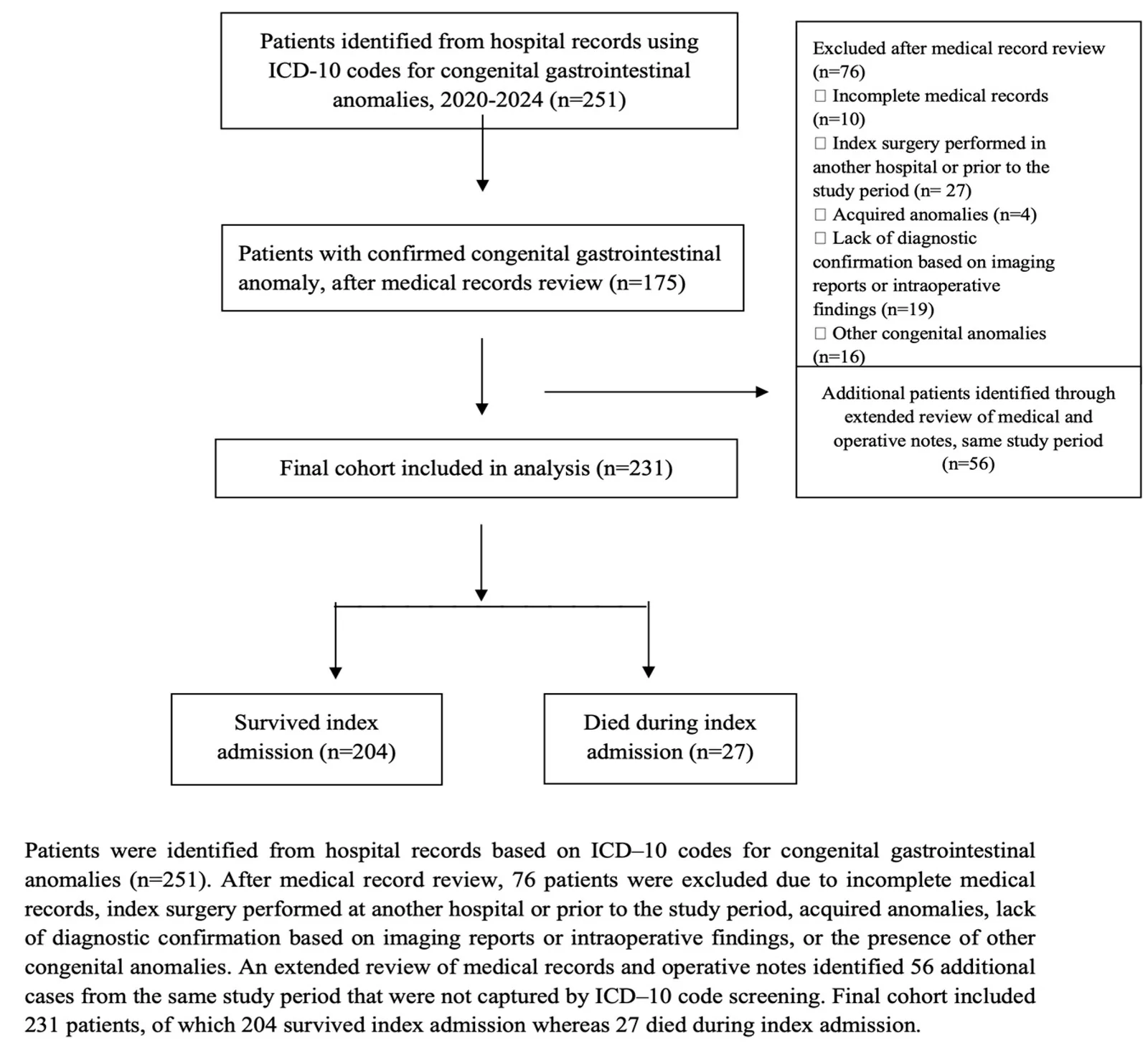
Flow diagram of patient selection and inclusion in descriptive and main mortality analysis.

Following institutional ethics approval, eligible cases were identified through screening of the institutional electronic medical database using International Classification of Diseases (ICD)-10 codes for congenital gastrointestinal anomalies [20]. This initial search yielded 251 cases, whose clinical and operative notes were manually reviewed to confirm eligibility. Of these, 175 patients met the inclusion criteria, and 76 were excluded. To reduce under-ascertainment related to administrative coding inaccuracies, we expanded manual screening of medical and surgical notes for the same study period and identified an additional 56 eligible patients. The final study cohort comprised 231 children (Figure 1).

No additional exclusions were made after eligibility was confirmed. Data were extracted from medical records available from the Pediatric Surgical Departments of the Emergency Hospital for Children “Saint Mary”, Iasi, by two independent reviewers using standardized data collection forms. Discrepancies were resolved by consensus.

Collected variables included demographic and perinatal data (age at admission, sex, county of residence, gestational age, birth weight, prematurity, mode of delivery, and Apgar scores), prenatal suspicion based on documented prenatal ultrasound, and maternal factors. We classified malformations by embryological segment (foregut, midgut, and hindgut) and noted any associated syndromes or non-gastrointestinal anomalies (cardiac defects, urogenital, etc.). Complex cases were defined as ≥2 gastrointestinal anomalies or a gastrointestinal anomaly associated with omphalocele/gastroschisis.

Perioperative details included timing (urgent/emergency vs. elective), anesthesia risk (ASA physical status classification), central venous catheterization, time from admission to surgery, operative access (open vs. minimally invasive), and surgical intervention (primary vs. staged procedure vs. diagnostic procedure). We also recorded secondary outcomes: ICU admission, length of hospital and ICU stay, enteral feeding achievement, and complications (surgical, medical, and sepsis).

Collected variables were established a priori and aligned with the published literature; for example, prematurity was <37 weeks’ gestation, and CGIMs were grouped into ten diagnostic categories. Patients were followed until discharge or in-hospital death, and primary outcome data was available for all patients. Variables collected for the study are provided in Supplementary Table S1.

### 2.4. Outcomes

The primary outcome was death during index admission, defined as death occurring during the first hospitalization related to a congenital gastrointestinal malformation. Secondary mortality outcomes included death occurring within 30 days of admission, death occurring after more than 30 days of admission, and 30-day postoperative mortality among patients who underwent surgery. Additional secondary outcomes included sepsis, length of hospital stay (LOS), duration of intensive care unit admission, requirement for central venous access, and achievement of full enteral feeding.

### 2.5. Definitions

The index admission was defined as the first hospital admission during the study period related to the index congenital gastrointestinal malformation and served as a reference admission for baseline outcome analysis. Subsequent admissions related to follow-up care, staged surgical management, or complications were recorded separately and were not treated as new cases.

For the purposes of the analysis, the primary congenital gastrointestinal malformations were grouped into ten diagnostic categories as follows: (1) esophageal atresia ± tracheoesophageal fistula; (2) duodenal atresia or stenosis; (3) hypertrophic pyloric stenosis; (4) intestinal malrotation +/− volvulus; (5) jejuno-ileal atresia or stenosis; (6) persistent omphalomesenteric duct remnants; (7) Hirschsprung disease; (8) cloacal malformation; (9) anorectal malformations; and (10) other gastrointestinal anomalies (e.g., duplication cyst, gastric volvulus).

When multiple CGIMs were present during the index admission, the index/primary diagnoses corresponded to the most proximal anatomical anomaly if diagnosed synchronously or the earliest confirmed diagnosis if diagnosed subsequently. Embryological segment was defined by the anatomical location of the congenital gastrointestinal malformations (foregut, midgut, or hindgut). When multiple malformations were present, classification was based on the most proximal lesion. The cases were classified as “complex” if they had at least two congenital gastrointestinal malformations identified during the study period or one eligible CGIM associated with an abdominal wall defect (omphalocele or gastroschisis). This composite definition was operationalized as a single binary variable and used in inferential statistics. In patients with complex CGIMs, the assigned diagnostic category reflected the index lesion.

Associated anomaly burden was defined as the number of associated non-gastrointestinal anomalies per patient. Syndromic status was defined as the presence of a recognized chromosomal disorder, genetic syndrome, developmental sequence, associations, or multiple congenital anomaly pattern accompanying the gastrointestinal malformations.

Sepsis was identified by retrospective review of medical records, including from physician documentation and discharge diagnoses recorded during index admission. Cases were classified as sepsis when the diagnosis was documented by the treating clinical team in the discharge record and was supported by compatible clinical findings and laboratory evidence of systemic infection documented in the medical record, when available. No independent adjudication or retrospective application of standardized pediatric sepsis criteria was performed. Because this was a retrospective study, complete application of a standardized pediatric sepsis definition could not be verified for every patient. Therefore, sepsis was analyzed as an exploratory in-hospital complication rather than a baseline predictor for mortality.

Prenatal ultrasound suspicion (hereafter, prenatal suspicion) was defined as documented prenatal ultrasonographic findings suggestive of a congenital gastrointestinal malformation that was subsequently confirmed postnatally by imaging and/or intraoperative findings.

American Society of Anesthesiologists (ASA) physical status was recorded from the preoperative anesthetic assessment and defined as the highest ASA Physical Status Classification during index admission immediately before the index surgical procedure. ASA classification was available for all operated patients (*n* = 216). Patients who did not undergo surgery were excluded from analyses involving ASA status.

### 2.6. Quality Control

Data was extracted by two reviewers using a standardized protocol and predefined data dictionary. Discrepancies were resolved by consensus and a random audit of approximately 10% of the records was performed to verify accuracy and completeness.

### 2.7. Bias

Selection bias was reduced through the consecutive inclusion of all eligible patients during the study period and supplementary manual case ascertainment. Information bias was minimized through standardized data abstraction, predefined variable definitions, and independent review. Prenatal suspicion was particularly vulnerable to under-ascertainment because external prenatal imaging reports were not consistently available for referred patients. This limitation may have resulted in non-differential misclassification and conservative estimates of prenatal detection. These limitations should be considered when interpreting findings from this tertiary referral cohort.

### 2.8. Statistical Analysis

Statistical analyses were performed using SPSS Statistics version 31 (IBM Corp., Armonk, NY, USA). Continuous variables are presented as mean (SD) or median (IQR), as appropriate. Categorical variables are reported as frequencies and percentages, with variable-specific denominators where applicable. Minimum and maximum values are reported for selected skewed variables. For unadjusted comparisons according to survival status at discharge from the index admission, continuous and ordinal variables were compared using the Mann–Whitney U test. Categorical variables were compared using Pearson’s χ^2^ test, Fisher’s exact test, or Fisher–Freeman–Halton exact test, as appropriate. Crude associations with mortality were quantified using odds ratios (ORs) with 95% confidence intervals (CIs).

To minimize overfitting, the primary multivariable logistic regression model was restricted to prespecified baseline covariates selected a priori based on clinical relevance, the previous literature, and the limited number of mortality events. The primary multivariable model included prematurity, embryological segment, and the presence of associated non-gastrointestinal anomalies. Because gestational age and prematurity were highly collinear, only prematurity (yes/no) was entered into the multivariable model.

Because prematurity and birth weight are closely related, birth weight was evaluated in a separate model rather than entered simultaneously with prematurity. Birth weight was analyzed as a continuous variable (per 1 kg increase). ASA physical status was not included in the primary multivariable model because it reflects preoperative illness severity and may lie on the causal pathway to mortality. Syndromic status and complex cases were not included in the primary model because these variables were considered markers of overall disease complexity that overlap conceptually with associated anomaly burden and lesion characteristics.

The primary adjusted analysis used complete-case data. Model calibration was assessed using the Hosmer–Lemeshow goodness-of-fit test, while discrimination was evaluated using the area under the receiver operating characteristic curve (AUC) derived from model-predicted probabilities. Interaction terms were not formally evaluated because the limited number of deaths provided insufficient statistical power for reliable interaction analyses.

The missing-data patterns were assessed using Little’s test for missing completely at random (MCAR), and variables with missing data are summarized in Supplementary Table S1. Missing baseline data were addressed in a prespecified sensitivity analysis using multiple imputations by fully conditional specification (FCS). Twenty imputed datasets were generated with 10 iterations per dataset. Continuous variables (gestational age, birth weight, Apgar score) were imputed using predictive mean matching with five nearest neighbors, whereas antenatal diagnosis of congenital gastrointestinal malformation was imputed using logistic regression. The imputation model included gestational age, birth weight, Apgar score, antenatal diagnosis, sex, embryological segment, any associated non-gastrointestinal anomalies, and the primary outcome (index-admission mortality). Gestational age was imputed as a continuous variable; following imputation, prematurity was derived from the imputed gestational age (<37 weeks’ gestation) and entered the multivariable sensitivity analysis. Inspection of the iteration history showed stable summary statistics across successive iterations, supporting adequate convergence of the FCS algorithm. Regression estimates were pooled across the 20 imputed datasets using Rubin’s rules. Sensitivity analysis also included substitution of birth weight for prematurity in the primary multivariable model and restriction of the cohort to neonates [21].

A secondary exploratory model evaluated the association between sepsis and mortality after adjustment for prematurity and embryological segment. Because sepsis occurred during admission and could lie on the causal pathway to death, it was analyzed separately from the primary model. Also, as sepsis was identified retrospectively from routine clinical documentation rather than by prospective application of standardized diagnostic criteria, this analysis was considered exploratory and intended to evaluate association rather than causation.

Maternal age was analyzed using three predefined groups (<20 years, 20–35 years, and >35 years), selected according to the established obstetric risk stratification criteria [22]. The 20–35 group served as the reference group. Temporal trends in mortality were assessed using the linear-by-linear association test. Admissions were also categorized into early (2020–2021) and late (2022–2024) pandemic periods. These analyses were exploratory. Exploratory cut-off values for continuous variables were derived from receiver operating characteristic (ROC) analyses using the Youden index and were not used for hypothesis testing or model construction. All statistical tests were two-sided, and *p* < 0.05 was considered statistically significant.

### 2.9. Ethical Considerations

Ethical approval was obtained from the Institutional Research and Ethics Committee of the Emergency Hospital for Children “Saint Mary”, Iasi, Romania (project identification code 18879; approval date: 6 June 2024). The Committee approved the retrospective review of medical records covering the predefined study period (1 January 2020 to 31 December 2024). Data extraction and analysis commenced only after ethics approval had been granted. The study was conducted in accordance with the Declaration of Helsinki and the conditions specified by the Institutional Research and Ethics Committee [23].

## 3. Results

### 3.1. Cohort Assembly and Baseline Characteristics

We identified 231 patients with CGIMs admitted over 5 years. Median admission age was 2 days (IQR 0–46); 136 (58.9%) were males. Median gestational age was 38 weeks (IQR 36–39 weeks) and the median birth weight was 2.9 kg (IQR 2.52–3.28 kg). Prematurity was present in 53/190 patients (27.9%). Overall, 27 (11.7%) patients died during the index admission. Table 1 compares baseline features by survival.

Compared with survivors, non-survivors had a significantly lower birth weight (median 2.53 vs. 3 kg, *p* < 0.001), gestational age (36 vs. 38 weeks, *p* = 0.001), and Apgar scores (7 vs. 8, *p* < 0.001), and a higher prevalence of prematurity (52% vs. 24.2%, *p* = 0.004). A higher proportion of non-survivors were female (66.7% vs. 37.7%, *p* = 0.004). Prenatal suspicion of CGIM was more common among non-survivors than survivors (33.3% vs. 15.7%, *p* = 0.033). Denominators vary due to missing data; see Supplementary Table S1.

### 3.2. Malformations Spectrum and Associated Anomalies

Foregut anomalies (esophageal atresia, etc.) accounted for 40.3% of cases, midgut anomalies (intestinal atresias, malrotation) for 35.5%, and hindgut anomalies (anorectal malformations, cloacal malformations, etc.) for 24.2%. The distribution of CGIM types is summarized in Table 2. Mortality varied by type: for example, esophageal atresia had a 33.3% death rate and duodenal atresia 34.5%, whereas some defects such as congenital hypertrophic pyloric stenosis, Hirschsprung disease, or omphalomesenteric duct remnants had 0% mortality (Table 2).

Complex cases were identified in 19 patients (8.2%) [18]. Associated non-gastrointestinal congenital anomalies were identified in 162 of 231 patients (70.1%).

Having a recognized syndromic or sequence-associated anomaly was much more common in non-survivors: 43.3% of children who died vs. 7.0% of survivors (*p* < 0.001). Cardiac defects were the most frequent associated anomalies (51.1% of those with any anomaly).

Syndromic status was identified in 30 patients (13%). Chromosomal abnormalities were the most common diagnoses, affecting 18 patients (7.8%), most commonly trisomy 21 that was concentrated among patients with duodenal atresia/stenosis. Recognized malformation sequences and associations were detected in six patients (2.6%), including VACTERL association (*n* = 5) and Pierre Robin sequence (*n* = 1). Rare additional syndromic conditions included achondroplasia, fetal alcohol syndrome, trisomy 18, and chromosomal 10q deletion syndrome, each observed in isolated cases. Three further patients (1.3%) presented with plurimal formative syndromes that remained without a specific diagnosis. The remaining 201 patients (87%) had no recognized syndromic or etiologic diagnosis.

### 3.3. Perioperative Management

Of 231 patients, 216 underwent surgery during the admission. Primary operative management was undertaken in 163 patients (70.6%) and staged procedures in 34 (14.7%); 19 patients (8.2%) had only diagnostic procedures. Operative management categories were not mutually exclusive; some complex cases underwent both primary and staged or diagnostic procedures. Emergency or urgent surgery was required in 202 (87.4%) patients.

The median time from admission to surgery was approximately 38 h (data available in 189 patients) and did not differ between survivors and non-survivors (38.19 vs. 38.90 h).

Open surgery was the predominant approach (188 patients, 81.4%), whereas minimally invasive surgery was completed in 24 patients (10.4%), with conversion from minimally invasive to open surgery in three cases (1.3%). Median operative time was 160 min (IQR 119–210).

Regarding anesthesia risk, most patients were classified as ASA III (63%), followed by ASA II (24.5%), ASA IV (10.4%), and ASA V (1.4%). When grouped into lower (ASA I–III) and higher (ASA IV–V) preoperative risk, 189 patients (87.5%) belonged to ASA I–III category, whereas 27 patients (12.5%) were classified as ASA IV–V.

### 3.4. Complications and Secondary Outcomes

**Figure 2:**
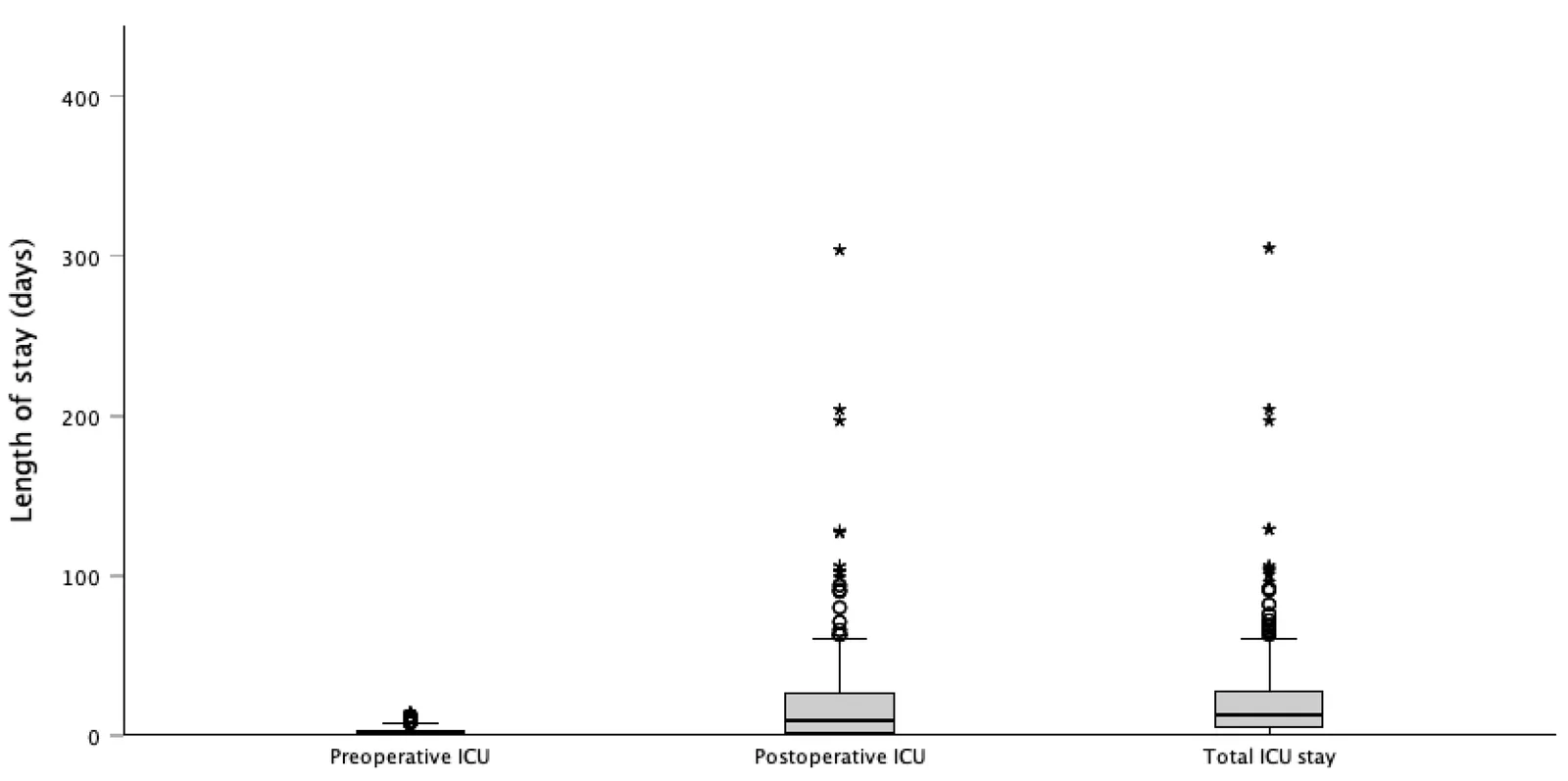
Distribution of intensive care unit (ICU) stay duration during the index admission. * Boxplots show median (horizontal line), interquartile range (box), and whiskers extending to 1.5× the interquartile ranges and outliers (circles and asterisks).

A high proportion of patients required intensive care: 90.5% (209/231) had at least one ICU admission (Figure 2). Median preoperative, postoperative and total ICU stay were 1, 9, and 13 days.

**Figure 3:**
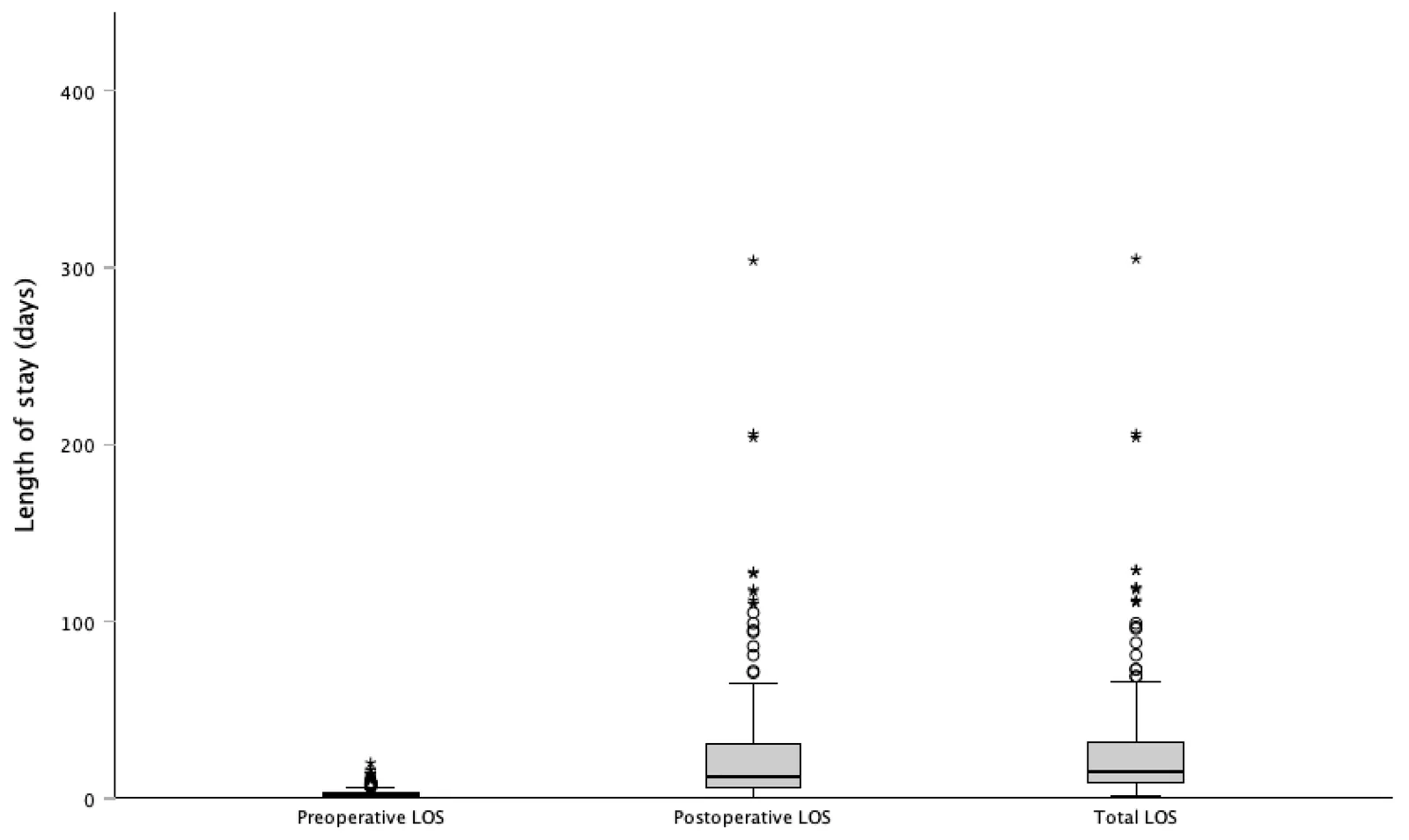
Distribution of length of stay (LOS) during the index admission. * Boxplots show median (horizontal line), interquartile range (box), and whiskers extending to 1.5× the interquartile ranges and outliers (circles and asterisks).

Length of stay was longer among non-survivors than survivors (median 25 days vs. 15 days, respectively), although the difference was of borderline statistical significance (*p* = 0.050). Median preoperative, postoperative, and total hospitalization during index admission were 2, 12, and 15 days. (Figure 3).

Central venous access was required in 177 patients (76.6%). All patients who died during index admission had central venous access.

Overall, complications occurred in 108 patients (46.8%): 28 patients (12.1%) had isolated surgical complications, 45 (19.5%) isolated medical complications, and 35 (15.2%) both. The most common surgical complications were anastomotic leak/fistula (25/63, 39.7%), bowel obstruction (15/63, 20.6%), wound dehiscence (15/63, 23.8%), and stoma-related complications (13/63, 20.6%). Sepsis occurred in 48 patients (20.8%). Children who developed sepsis had substantially higher mortality (39.6% vs. 4.4%, chi-square = 45.676, *p* = 0.001). Surgical complications were also associated with higher mortality; 25.4% (16/63) of patients with surgical complications died, compared with 6.5% (11/168) without surgical complications.

Enteral feeding was initiated later in non-survivors than survivors (median 5.5 days [IQR 5–6] vs. 4 days [IQR 2–6]). Similarly, time to full enteral feeding was longer among non-survivors (median 17.5 days [IQR 12–23] vs. 6 days [IQR 3–18]).

### 3.5. Mortality Analyses

Mortality during index admission occurred in 27 of 231 patients (11.7%). Among 27 deaths, 15 (55.6%) occurred within 30 days of admission and four patients (14.8%) died before undergoing surgery. The four preoperative deaths comprised two patients with esophageal atresia ± tracheoesophageal fistula and two duodenal atresia/stenosis. Three patients were born prematurely, one had trisomy 21 associated with complex congenital heart disease, and all deteriorated rapidly before surgery. Individual patient characteristics are presented in Supplementary Table S4.

The median age at death was 27 days of life (IQR 14–48). Among the 23 postoperative deaths (10.6% of the total cohort), 11 occurred within 30 days after surgery, whereas 12 occurred beyond 30 days postoperatively. Mortality was notably higher in patients with any associated non-gastrointestinal anomalies (88.9% vs. 67.6%, χ^2^ test = 5.14, *p* = 0.023). Mortality was also higher in children with lower gestational age and birth weight; 52% of non-survivors were premature vs. 24.2% of survivors (*p* = 0.004). Female patients accounted for 66.7% (18/27) of deaths despite representing 41.1% (95/231) of the cohort, whereas males accounted for 33.33% (9/27) of deaths while comprising 58.9% of the cohort. The distribution of sex and mortality across diagnostic categories is presented in Supplementary Table S3. Excluding patients with cloacal malformations, which occurred exclusively in females, did not materially change the univariate association between sex and mortality (male vs. female: OR 0.332, 95% CI 0.140–0.789, *p* = 0.013).

Surgical approach was not associated with in-hospital mortality. Open surgery was the predominant approach in both survivors and non-survivors (87%), with minimally invasive approaches accounting for 13% of procedures in each group. Open surgery was the predominant approach among patients who died both within 30 days after admission and beyond (90.9% vs. 83.3%). In univariable analysis (Table 3), the following variables were significantly associated with index-admission mortality: prematurity (gestational age < 37 weeks), Apgar score, birth weight, prenatal suspicion, ASA status, embryological segment, presence of any non-gastrointestinal anomaly, complex cases, syndromic status, female sex, and sepsis (all *p* < 0.05). Prematurity (OR 3.39, 95% CI 1.43–8.01, *p* = 0.006), syndromic status (OR 10.21, 95% CI 4.14–25.21), complex CGIM (OR 5.6, 95% CI 1.98–15.84), and preoperative ASA IV–V status (OR 12.95, 95% CI 4.89–34.26) were associated with increase odds of index-admission mortality.

No statistically significant associations were observed for maternal age (overall Wald test, *p* = 0.937), pregnancy type, pandemic phases or temporal trends across the study period. Mortality rates were 14.6%, 13.3%, 9.1%, 10.9%, and 11.4% in 2020–2024. Although syndromic and complex cases were strongly associated with mortality in univariable analyses, these variables were not included in the primary multivariable model because of concerns regarding overfitting and conceptual overlap with other measures of disease complexity.

In the primary multivariable logistic regression (190 patients, 25 deaths), adjusted for embryological segment and associated non-gastrointestinal anomalies, prematurity remained the strongest independent predictor associated of mortality (adjusted OR 3.20, 95% CI 1.23–8.31, *p* = 0.017). Embryological segment was also independently associated with mortality (overall *p* = 0.008). Compared with foregut anomalies, patients with midgut anomalies had lower odds of mortality (adjusted OR 0.06, 95% CI 0.008–0.48, *p* = 0.008), whereas the association for hindgut anomalies did not reach statistical significance (adjusted OR 0.29, 95% CI 0.08–1.09, *p* = 0.068). The presence of associated non-gastrointestinal anomalies was not independently associated with mortality (adjusted OR 2.33, 95% CI 0.63–8.66, *p* = 0.208). The model showed adequate calibration (Hosmer–Lemeshow chi-square = 3.06, df = 6, *p* = 0.802), and acceptable discrimination (AUC = 0.793).

Missing data were present for several baseline variables (Supplementary Table S1), including gestational age (17.7%), birth weight (19.9%), Apgar score (23.4%) and antenatal diagnosis (17.7%) (Supplementary Table S1). Little’s MCAR test did not provide evidence against the assumption that the data were missing completely at random (chi-square = 47.81, df = 35, *p* = 0.073).

Sensitivity analyses, including multiple imputation of incomplete baseline variables, substitution of birth weight for prematurity, and restriction to neonates, yielded findings broadly consistent with the primary model (Supplementary Table S2). After pooling estimates across the 20 imputed datasets using Rubin’s rules, prematurity remained independently associated with index-admission mortality (adjusted OR 3.32, 95% CI 1.27–8.74, *p* = 0.014). Compared with foregut anomalies, patients with midgut anomalies had lower odds of death (adjusted OR 0.09, 95% CI 0.02–0.42, *p* = 0.002), whereas the association for hindgut anomalies did not reach statistical significance (adjusted OR 0.28, 95% CI 0.07–1.03, *p* = 0.055). Associated non-gastrointestinal anomalies were not independently associated with mortality (adjusted OR 3.14, 95% CI 0.86–11.48, *p* = 0.084). Similar results were observed in the neonates-only analysis (adjusted OR 3.32, 95% CI 1.24–8.90; *p* = 0.017). In an alternative model in which birth weight replaced prematurity, lower birth weight was independently associated with mortality (adjusted OR 0.34, 95% CI 0.16–0.74; *p* = 0.006). Overall, sensitivity analyses produced findings consistent with the primary analysis.

Because sepsis represents an in-hospital complication rather than a baseline characteristic, it was evaluated separately in an exploratory analysis. Patients with sepsis had substantially higher mortality than those without sepsis (39.6% vs. 4.4%, *p* < 0.001). After adjustment for prematurity and embryological segment, sepsis remained strongly associated with mortality (adjusted OR 8.63, 95% CI 3.16–23.55; *p* < 0.001). Given the retrospective ascertainment of sepsis, the limited number of deaths, and the exploratory nature of this analysis, these findings should be interpreted cautiously.

Mortality rates remained relatively stable throughout the study period, ranging from 9.1% to 14.6%. No significant temporal trend was observed (linear-by-linear association chi-square = 0.326, *p* = 0.568). Receiver operating characteristic (ROC) analysis showed moderate discrimination for mortality for the Apgar score (AUC 0.770), age at admission (AUC 0.753), birth weight (AUC 0.723), and gestational age (AUC 0.697). The optimal cut-off values identified using the Youden index were an Apgar score less than 7, age at admission < 4.5 days, birth weight < 2.08 kg, and gestational age < 37.5 weeks (Table 4).

## 4. Discussion

In this outborn tertiary referral cohort, approximately one in nine children with CGIM died during the index admission. Mortality was concentrated among neonates and was highest in patients with foregut malformations, particularly esophageal atresia ± tracheoesophageal fistula and duodenal atresia/stenosis. Prematurity remained independently associated with mortality, while sepsis emerged as the strongest in-hospital correlate of death. These results suggest that early biological vulnerability and infection-related factors during admission critically compromise survival in this setting. Our mortality rate is higher than that reported in many Western series, possibly reflecting differences in patient severity, referral pathways, and available resources [2,9,24].

Mortality among children with congenital gastrointestinal malformations varies widely between healthcare systems [2,25]. In high-income countries, mortality in children with congenital gastrointestinal malformations ranges from 1 to 10%, whereas studies from low-income settings report rates between 30 and 70% or more [8,26,27]. The mortality observed in our cohort (11.7%) lies between these extremes and likely reflects challenges of managing a high-risk referral population in a resource-limited setting. As previously reported, we found that prematurity, low birth weight, and the associated anomaly increase risk are important markers of mortality risk [17,28,29]. Mortality was particularly high among patients with foregut malformations, especially esophageal atresia and duodenal atresia/stenosis, consistent with previous reports [8,30,31].

The longer hospital stay observed among non-survivors should be interpreted cautiously because in patients who died during prolonged admissions, hospital length of stay primarily reflects time-to-death rather than resource utilization comparable with that of survivors.

Female sex was associated with higher mortality in the univariable analysis. This finding should be interpreted cautiously because deaths were concentrated within a small number of diagnostic categories, including cloacal malformations, which occurred exclusively in females. However, the association persisted after the exclusion of patients with cloacal malformations. In addition, chromosomal anomalies were predominantly observed with specific diagnostic categories and may have contributed to differences in disease complexity. Because several diagnostic categories contained few or no deaths, reliable adjustment for diagnostic categories was not feasible, as the exploratory model did not converge. Therefore, residual confounding by diagnostic category cannot be excluded.

Trisomy 21 occurred predominantly within the duodenal atresia/stenosis group, consistent with the well-established findings reported in the published literature [32]. Additionally, the strong effect of associated anomalies supports the need for early multidisciplinary assessment, particularly when gastrointestinal malformations coexist with chromosomal or multisystem disorders, especially cardiac defects [33].

Premature patients have reduced physiological reserve, are more vulnerable to postoperative complications, and often present additional challenges in perioperative stabilization and nutritional support [17,28,34,35].

Sepsis greatly amplified the risk; nearly one third of septic patients died [36,37,38]. This highlights the need for aggressive infection control and early sepsis management protocols. The observed association should be interpreted cautiously. Sepsis was identified retrospectively from routine clinical documentation and discharge records rather than by prospective application of standardized pediatric sepsis criteria. Consequently, some degree of misclassification cannot be excluded, and the exploratory analysis should be regarded as hypothesis-generating rather than evidence of an independent causal relationship. Several contextual factors may contribute. Many patients lived in rural areas (70.1%) and may have faced transport delays. Our healthcare workers, ICU and surgical resources, though substantial, may be stretched, especially during COVID-19 pandemic years [39]. Compared with Western registries, limited neonatal intensive care availability and higher infection prevalence may underlie our higher mortality [40].

Prenatal documentation was incomplete and depended on the transfer records from external centers, which introduces substantial scope for misclassification. Moreover, lesions that are more severe and more readily visible on antenatal imaging are also more severe and more likely associated with adverse outcomes. Prenatal suspicion in this cohort is therefore unlikely to represent a protective or harmful factor itself; instead, it probably acts as a marker of lesion severity and documentation quality. This is, to our knowledge, the largest single-center Romanian CGIM series reporting data on mortality. We included all cases over five years and used robust statistical methods.

The study has several limitations. The retrospective design and the presence of missing data may have introduced bias. In addition, the cohort was derived from a single tertiary center and consisted entirely of transferred (outborn) patients, which may have enriched the population for more complex cases and critically ill neonates, limiting the generalizability of the findings to other clinical settings. The study also included a heterogeneous spectrum of congenital anomalies, encompassing malformations with substantially different disease severity, management pathways, and baseline mortality risks. Although adjustment for embryological segment partially accounted for this variability, the limited number of deaths within the individual diagnostic categories restricted diagnosis-specific analyses and reduced the precision of certain estimates. Also, the relatively small number of outcome events restricted multivariable modeling and limited the ability to detect weaker independent associations. Although multiple imputation was used to reduce potential bias arising from incomplete baseline data, its validity depends on the missing-at-random assumption and correct specification of the imputation model. Nevertheless, the close agreement between complete-case and multiply imputed analyses suggests that missing baseline data did not materially influence the primary conclusions.

Finally, these findings should be interpreted within the context of an outborn tertiary referral population and may not be directly generalizable to population-based cohorts or inborn neonatal surgical centers.

Premature neonates with CGIMs should be recognized as high-risk. They may benefit from heightened surveillance and earlier intervention. Given the impact of sepsis, implementing early-warning systems and strict infection control in the neonatal surgical ward is warranted. Improving prenatal detection and referral pathways might also improve outcomes.

## 5. Conclusions

Mortality during index admission remained substantial among children with congenital gastrointestinal malformations treated at this tertiary referral center. Prematurity was the only baseline factor independently associated with mortality, whereas retrospectively identified sepsis was associated with fatal outcomes in exploratory analyses. These findings highlight the need to optimize care for preterm infants and strengthen the prevention and early management of severe infection during admission. Because sepsis was identified retrospectively and without standardized diagnostic criteria, this association should be confirmed in prospective multicenter studies. Additional multicenter studies in Eastern Europe would help validate these findings and refine risk-stratification strategies for high-risk patients across different healthcare settings.

## Figures and Tables

**Table 1:** Baseline demographic and perinatal characteristics according to survival status at index-admission discharge.

Variable	Survivors (*n* = 204 **)	Non-Survivors (*n* = 27 **)	*p*-Value
Age at admission (days)	3.5 (0–56)	0 (0–1)	<0.001
Apgar score	8 (8–9)	7 (6–8)	<0.001
Birth weight, kg	3.00 (2.59–3.30)	2.53 (1.84–2.97)	<0.001
Female sex, *n* (%)	77 (37.7)	18 (66.7)	0.004
Gestational age, weeks	38 (37–39)	36 (33.5–38)	0.001
Maternal age, years	29 (24–34)	27 (22–35)	0.942
Maternal comorbidities, *n* (%)	43/159 (27.0)	5/21 (23.8)	0.753
Multiple pregnancy, *n* (%)	6/193 (3.1)	1/27 (3.7)	1.000 *
Prematurity, *n* (%)	40/165 (24.2)	13/25 (52.0)	0.004
Prenatal suspicion of congenital gastrointestinal malformation, *n* (%)	32 (15.7)	9 (33.3)	0.033 *
Rural residence, *n* (%)	136 (66.7)	20 (74.1)	0.440

* Fisher’s exact test. ** Data are presented as median (Q1–Q3) or *n* (%). Variable specific denominators are reported where data is incomplete. Missing data for all variables is summarized in Supplementary Table S1.

**Table 2:** Mortality according to congenital gastrointestinal malformation type.

Diagnostic	Survivors	Non-Survivors	Total
Esophageal atresia ± tracheoesophageal fistula	24 (66.7%)	12 (33.3%)	36
Duodenal atresia/stenosis	19 (65.5%)	10 (34.5%)	29
Congenital hypertrophic stenosis	27 (100.0%)	0 (0.0%)	27
Intestinal malrotation	18 (100.0%)	0 (0.0%)	18
Jejunoileal atresia/stenosis	23 (92.0%)	2 (8.0%)	25
Omphalomesenteric duct remnants	37 (100.0%)	0 (0.0%)	37
Hirschsprung disease	20 (100.0%)	0 (0.0%)	20
Cloacal malformations	2 (50%)	2 (50.0%)	4
Anorectal malformations	31 (96.9%)	1 (3.1%)	32
Others	3 (100.0%)	0 (0.0%)	3
**TOTAL**	**204 (88.3%)**	**27 (11.7%)**	**231**

Footnote: Data are presented as *n* (%). Fisher–Freeman–Halton exact test: *p* < 0.001.

**Table 3:** Factors associated with mortality during index admission—univariable logistic regression analysis.

Variable	Comparison	OR	95% CI	*p*-Value
APGAR score	per 1-point increase	0.536	0.388–0.742	<0.001
ASA status	preoperative ASA IV-V vs. ASA I-III	12.95	4.89–34.26	<0.001
birth weight	per 1 kg increase	0.243	0.114–0.515	<0.001
embryological segment	midgut vs. foregut	0.081	0.018–0.355	<0.001
	hindgut vs. foregut	0.183	0.052–0.643	0.008
pandemic phases	late vs. early	0.712	0.316–1.601	0.411
prematurity	yes vs. no	3.385	1.430–8.013	0.006
prenatal suspicion of CGIM	yes vs. no	3.259	1.387–7.660	0.006
pregnancy type	multiple vs. singleton	1.199	0.139–10.357	0.869
sex	male vs. female	0.303	0.130–0.708	0.006
syndromic status	yes vs. no	10.21	4.14–25.21	<0.001
type of CGIM	complex vs. single CGIM	5.600	1.980–15.840	0.001

Note: Odds ratios were obtained from univariable binary logistic regression analyses evaluating factors associated with mortality during index admission.

**Table 4:** Receiver operating characteristic (ROC) analysis of clinical predictors of mortality during the index admission.

Variable	AUC (95% CI)	*p*-Values	Optimal Cut-Off	Sensitivity (%)	Specificity (%)
Age at admission (days)	0.753 (0.679–0.828)	<0.001	≤4.5	96.3	48.5
Apgar score	0.770 (0.662–0.878)	<0.001	≤7	59.1	83.2
Birth weight (kg)	0.723 (0.617–0.829)	<0.001	≤2.08	41.7	91.9
Gestational age (weeks)	0.697 (0.580–0.815)	<0.001	≤37.5	64.0	71.1

Footnote: AUC, area under curve; CI, confidence interval. Optimal cut-off values were determined using the Youden index.

## Data Availability

The data presented in this study are available on request from the corresponding author. The data are not publicly available due to privacy and ethical reasons.

## Supplementary Materials

The following supporting information can be downloaded at https://www.mdpi.com/article/10.3390/children13080976/s1. Table S1. Missing data across variables included in descriptive and regression analysis. Table S2. Sensitivity analyses of factors independently associated with mortality during index admission. Table S3. Sex distribution and mortality according to congenital gastrointestinal malformation category. Table S4. Characteristics of patients who died before undergoing surgery.

## Abbreviations

The following abbreviations are used in this manuscript:

ASA	American Society of Anesthesiologists
CGIM	Congenital gastrointestinal malformation
ICU	Intensive care unit
IQR	Interquartile range
χ^2^	Chi-square
MI	Multiple imputation
OR	Odds ratio
ICD–10	International Classification of Diseases, 10th Revision
VACTERL	Vertebral, anal, cardiac, tracheoesophageal fistula, renal, limb anomalies
CI	Confidence interval
LOS	Length of hospital stay
ROC	Receiver operating characteristic
AUC	Area under curve
MCAR	Missing completely at random
STROBE	Strengthening the Reporting of Observational Studies in Epidemiology
SPSS	Statistical Package for the Social Sciences (or IBM SPSS Statistics)
TEF	Tracheoesophageal fistula
Q1–Q3	First quartile–third quartile
