# Supplementary material for: Mortality and Factors Associated with Death in Children with Congenital Gastrointestinal Malformations—A Five-Year Retrospective Study from Romania (2020–2024)
Source: Children (Basel). 2026 Jul 23;13(8):976. doi: 10.3390/children13080976 (PMC13511268; doi:10.3390/children13080976)
Supplement: Supplementary file 1 [file children-13-00976-s001.zip › children-4409044-supplementary.pdf]

Supplementary Table S1 – Missing data across variables included in descriptive and regression analysis*

Variable	Available	Missing (n)	Missing (%)
Apgar score	177	54	23,4
Birth weight	185	46	19,9
Delivery mode	189	42	18,2
Gestational age	190	41	17,7
Maternal age	169	62	26,8
Maternal comorbidities	180	51	22,1
Pregnancy type	220	11	4,8
Prenatal ultrasound	190	41	17,7
Time to full enteтral feeding	194	37	16
Time to start enteral feeding	207	24	10,4
Time to surgery	189	42	18,2

* Only variables with missing data are shown. Variables with complete data and derived variables sharing identical missingness patterns with source variables were not presented.

Supplementary Table S2. Sensitivity analyses of factors independently associated with mortality during index admission

Variable	Primary complete-case model (n=190), adjusted OR (95%CI), p-value	Multiple imputation model (20 imputations, n=231), adjusted OR (95%CI), p-value	Birth weight substitution model (n=185), adjusted OR (95%CI), p-value	Neonatal only model, adjusted OR (95%CI), p-value
Prematurity	3.20 (1.23-8.31), p=0.017	3.22 (1.24-8.36), p=0.015	-	3.32 (1.24-8.90), p=0.017
Birth weight (kg)	-	-	0.34 (0.16-0.74), p=0.006	-
Midgut vs foregut	0.06 (0.008-0.48), p=0.008	0.09 (0.02-0.42), p=0.002	0.08 (0.01-0.59), p=0.014	0.06 (0.007-0.46), p=0.007
Hindgut vs foregut	0.29 (0.08-1.09), p=0.068	0.27 (0.07-1.00), p=0.052	0.21 (0.04-0.97), p=0.045	0.25 (0.07-0.94), p=0.041
Associated non-gastrointestinal anomaly	2.33 (0.63-8.66), p=0.208	3.10 (0.84-11.46), p=0.087	1.86 (0.48-7.23), p=0.373	1.40 (0.35-5.62), p=0.631

Abbreviations: OR, odds ratio; CI , confidence interval

Footnote: The primary model adjusted for prematurity, embryological segment, and associated non-gastrointestinal anomalies. Multiple-imputation analyses were performed using chained equations with 20 imputations, and pooled estimates were combined according to Rubin's rules. The birth-weight substitution model replaced prematurity with birth weight because of the close biological relationship between gestational age and birth weight. The neonatal-only model was restricted to patients presenting during neonatal period.

Supplementary Table S3 – Sex distribution and mortality according to congenital gastrointestinal malformation category

**Diagnostic**	**Female patients, n**	**Female deaths, n (%)**	**Male patients, n**	**Male deaths, n (%)**
Esophageal atresia +/- tracheoesophageal fistula	20	7 (35)	16	5 (31.3)
Duodenal atresia/stenosis	16	6 (37.5)	13	4 (30.8)
Congenital hypertrophic stenosis	5	0 (0)	22	0 (0)
Intestinal malrotation	7	0 (0)	11	0 (0)
Jejunoileal atresia/stenosis	16	2 (12.5)	9	0 (0)
Omphalomesenteric duct remnants	7	0 (0)	30	0 (0)
Hirschsprung disease	6	0 (0)	14	0 (0)
Cloacal malformations	4	2 (50%)	0	0 (0)
Anorectal malformations	13	1 (7.7)	19	0 (0)
Others	1	0 (0)	2	0 (0)
**Total**	**95**	**18 (18.9)**	**136**	**9 (6.6)**

Supplementary Table S4 – Characteristics of patients who died before undergoing surgery

**Patient**	**Malformation category**	**Gestational age (weeks)**	**Associated anomalies**	**Reason for non-operative management**
Patient 1	Duodenal atresia/stenosis	31	Trisomy 21, complex congenital heart disease, severe acute kidney injury	Rapid clinical deterioration with multisystem organ failure
Patient 2	Esophageal atresia +/- tracheoesophageal fistula	29	Severe respiratory distress	Profound cardiorespiratory instability

Patient 3	Esophageal atresia +/- tracheoesophageal fistula	37	Severe congenital neurological anomalies (generalized cerebral atrophy, agenesis of the corpus callosum, microcephaly, generalised seizures) and severe hypoxic-ischemic injury at birth	Rapid neurological and cardiorespiratory deterioration
Patient 4	Duodenal atresia/stenosis	34	Severe respiratory distress	Cardiorespiratory arrest during induction of anesthesia
